# Design and Mechanical Sensitivity Analysis of a MEMS Tuning Fork Gyroscope with an Anchored Leverage Mechanism

**DOI:** 10.3390/s19163455

**Published:** 2019-08-07

**Authors:** Zezhang Li, Shiqiao Gao, Lei Jin, Haipeng Liu, Yanwei Guan, Shigang Peng

**Affiliations:** 1 State Key Laboratory of Explosion Science and Technology, Beijing Institute of Technology, Beijing 100081, China; 2 School of Mechatronical Engineering, Beijing Institute of Technology, Beijing 100081, China; 3 Beijing Institute of Control and Electronic Technology, Beijing 100038, China

**Keywords:** mechanical sensitivity, tuning fork gyroscope, anchored leverage mechanism, stiffness ratio, coordinate transformation method

## Abstract

This paper presents the design and analysis of a new micro-electro-mechanical system (MEMS) tuning fork gyroscope (TFG), which can effectively improve the mechanical sensitivity of the gyroscope sense-mode by the designed leverage mechanism. A micromachined TFG with an anchored leverage mechanism is designed. The dynamics and mechanical sensitivity of the design are theoretically analyzed. The improvement rate of mechanical sensitivity (IRMS) is introduced to represent the optimization effect of the new structure compared with the conventional one. The analytical solutions illustrate that the IRMS monotonically increases with increased stiffness ratio of the power arm (SRPA) but decreases with increased stiffness ratio of the resistance arm (SRRA). Therefore, three types of gyro structures with different stiffness ratios are designed. The mechanical sensitivities increased by 79.10%, 81.33% and 68.06% by theoretical calculation. Additionally, FEM simulation demonstrates that the mechanical sensitivity of the design is in accord with theoretical results. The linearity of design is analyzed, too. Consequently, the proposed new anchored leverage mechanism TFG offers a higher displacement output of sense mode to improve the mechanical sensitivity.

## 1. Introduction

A micro-electro-mechanical system (MEMS) gyroscope is a kind of inertial sensor used to detect the attitude angle and angular rate. It is based on an energy conversion of two vibrational modes due to the Coriolis effect [1,2]. With the rapid development of MEMS technology, the MEMS gyroscope has long been considered as an attractive and dynamic inertial sensor for a wide variety of applications, including the automotive industry, robotics, consumer electronics and high-volume military uses (0.1 to 100°/h). Compared with conventional gyroscopes, MEMS gyroscopes have many advantages, such as tiny volume, light weight, low power consumption, low cost, and the possibility of batch fabrication [3,4].

A common type of MEMS vibratory gyroscope is implemented as a tuning fork gyroscope (TFG). It is composed of two identical tines and two coupling mechanisms for synchronization of the anti-phase drive mode and anti-phase sense mode. The advantage of the TFG is that it can cancel the external common mode effect by applying a differential Coriolis detection between the two tines [5,6,7,8,9]. The sensitivity of the TFG is the biggest challenge in terms of superior performance.

The sensitivity of a micromachined TFG depends on two factors, peripheral circuit gain and the gyro’s own mechanical sensitivity. Much effort has already been made to improve the circuit gain and precision, and many achievements have been obtained [10,11]. Improving the sensitivity of the system only by enlarging the amplification of the peripheral circuit is meaningless. Since the Coriolis signal and the noise are often amplified at the same time, the system could not improve the signal-to-noise ratio (SNR). Therefore, to improve the sensitivity of vibratory gyroscopes, it is essential to achieve high mechanical sensitivity. The vibrational amplitude greatly improves when the work frequency equals the resonant frequency. Therefore, the displacement of differential tines is maximized and the mechanical sensitivity can be effectively increased when the two work modes of the gyroscope have the same resonant frequency (i.e., they are mode matched) [12,13]. It is difficult to completely match the resonant frequencies of the two modes through structural design due to fabrication imperfections. Therefore, mode matching through electrostatic tuning is often adopted [14,15,16,17,18]. Minimizing substrate energy dissipation and vacuum packaging can improve sensitivity owing to a high quality factor [19,20,21,22]. On the other hand, many studies on vibration type angular rate sensors in materials group them into bulk Si and polycrystalline Si [23]; some researchers focus on quartz gyroscopes based on material characteristics [24].

Amplification mechanisms are gaining importance in MEMS devices where motion reliability, precision, and sensitivity are needed. Researchers all over the world have proposed different types of amplification mechanisms, such as bridge-type, lever-type and four-bar linkage mechanisms [25,26,27,28,29,30]. The amplification mechanism has been applied to MEMS resonant output gyroscope (ROG) and MEMS accelerometer due to the force or displacement amplification effects [31,32]. The focus of this paper is on how to improve the mechanical sensitivity of a MEMS gyroscope by using a leverage mechanism.

In this paper, a new kind of micromachined TFG with an anchored leverage mechanism is proposed to analyze mechanical sensitivity. A conventional one with direct connection between sense-mode frame and proof mass is introduced at the same time for comparison. Detailed descriptions of the two design structures are provided in Section 2. Section 3 establishes and solves the dynamic equations for the response of the two architectures. FEM simulation of the stiffness of different springs on sense-mode and comparisons between simulation and analytical solutions are discussed and the linearity of the design is analyzed in Section 4. In Section 5, the discussion is given. Section 6 concludes the paper with a summary.

## 2. Architecture Design

**Figure 1:**
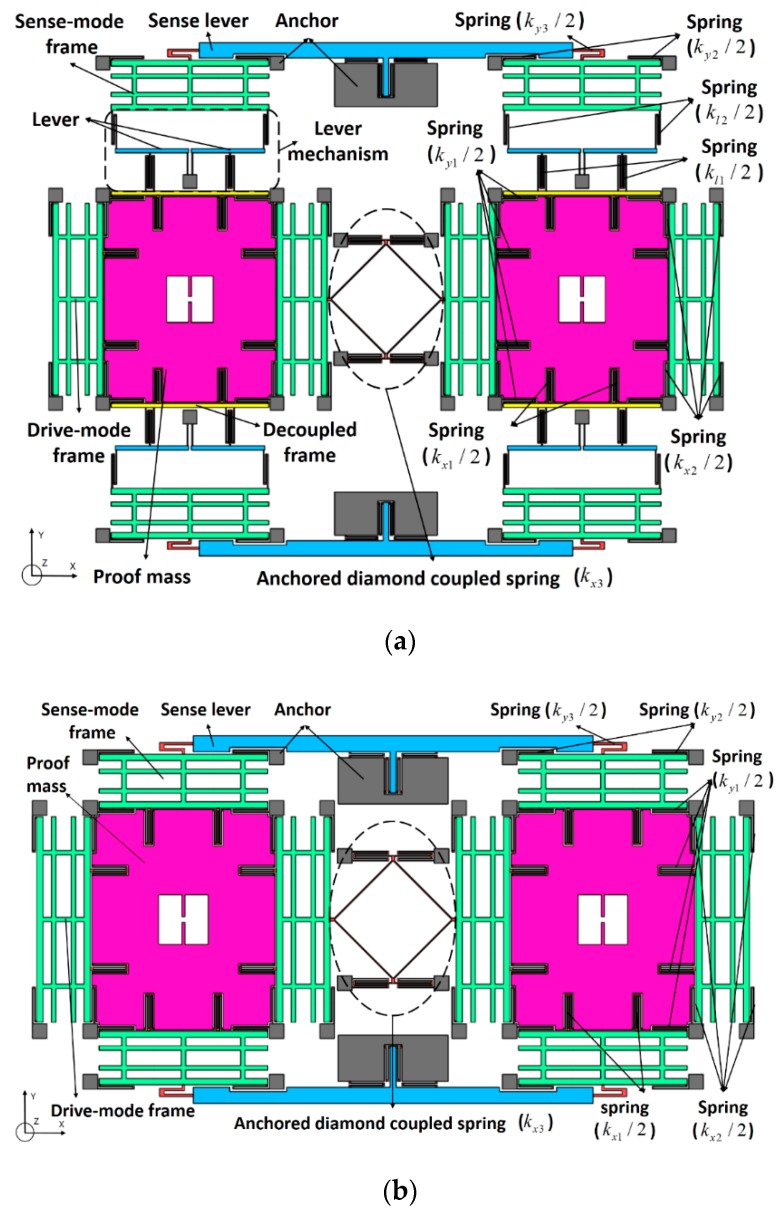
Schematic of designed tuning fork gyroscopes (TFGs): (**a**) type A, (**b**) type B.

In this paper, a novel MEMS tuning fork gyroscope with an anchored leverage mechanism is designed. The architecture of type A, as discussed in detail in Figure 1a, is a dual-mass structure that consists of an anchored diamond coupled spring, two sense levers, and two identical tines. As a conventional structure, type B is the same as type A, except for the connection between the proof mass and the sense-mode frame, as depicted in Figure 1b.

Both type A and type B are completely symmetrical double structured–decoupled architectures. Each tine contains a proof mass, two drive-mode frames and two sense-mode frames supported by symmetrical springs, and type A has an additional lever mechanism. In order to improve the robustness of the mode match between drive mode and sense mode, the springs are located on the same axis and can resist any temperature change of the resonance frequency, except the supporting lever springs and the anchored diamond coupled spring. All springs in the design are U-shaped in series or in parallel, which can reduce their axial stress to a greater extent, so that there is a linear relationship between force and displacement, which matches the electrodes by the variable-area capacitance mechanism.

## 3. Theoretical Analysis

### 3.1. Kinematic Analysis of Type A and B

The spring in Figure 1 represents the stiffness of the drive beams, the sense beams and the decoupling beams in the design of this paper. The stiffness of these beams is much less than that of the lever, frame and proof mass. On the other hand, the mass of the lever, frame, and proof mass is much larger than that of the beam. Therefore, the lever, frame and proof mass are simplified to inelastic mass and beam to massless spring. It is worth mention here that the lumped-parameter model used in this study is based on linear elastic material behavior, which is very widely used in the research of TFG [6,14,21,33,34,35]. Furthermore, the nonlinear analysis of the overall structure will be discussed in detail later. However, when the stiffness and mass of the beam are relatively large compared to other structures, some errors will occur and will not be adapted to this assumption.

**Figure 2:**
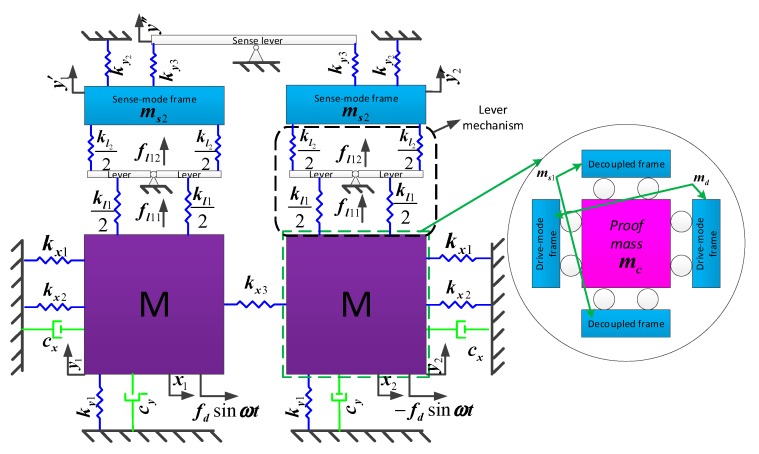
Model of type A.

According to Figure 1a, we can obtain the four-degrees-of-freedom (4-dof) coupling vibration model of the MEMS tuning fork gyroscope with an anchored leverage mechanism, as illustrated in Figure 2.

In operation, two proof masses and their respective drive mechanisms are electrostatically driven into anti-phase motion with the same amplitude along the drive direction by driving voltages imposed across the differential lateral comb electrodes on the drive mechanism. When an angular rate Ω*_z_* is applied, the anti-phase Coriolis acceleration of the proof mass induces linear anti-phase motions that are capacitively detected using differential parallel plate electrodes on the sense mechanism along the *y*-axis. The input angular rate Ω*_z_* can be calculated by the differential output.

Ideally, the structure is entirely symmetrical. Then, the two proof masses systems and damping coefficients are equal. The dynamics in the direction of sense mode are governed by the following:

Proof mass:

(1) $$\left\{ \begin{array}{l} (m_{s1}+m_{c}){\ddot{y}}_{1}+c_{y}{\dot{y}}_{1}+\left(k_{y1}+k_{l1}\right)y_{1}+f_{l11}=-2m_{c}\Omega_{z}{\dot{x}}_{1}\\ (m_{s1}+m_{c}){\ddot{y}}_{2}+c_{y}{\dot{y}}_{2}+\left(k_{y1}+k_{l1}\right)y_{2}+f_{l21}=-2m_{c}\Omega_{z}{\dot{x}}_{2} \end{array}\right.$$

where $m_{c}$, $m_{s1}$ and $m_{s2}$ are the mass of the proof mass, decoupled frame and sense-mode frame, respectively. $c_{y}$ is the damping coefficient of each tine in sense direction. $k_{y1}$ is the stiffness of the spring connected to $m_{c}$ and the drive-mode frame. $k_{l1}$ represents the stiffness of the spring connected to decoupled frame and lever in the sense direction. $f_{l11}$ and $f_{l11}$ represent the force exerted on the lever by the decoupled frame in the left and right tines. Ω*_z_* is the angular rate and the drive velocity ${\dot{x}}_{1}$ can be defined as:

(2) $$\{\begin{array}{c} {\dot{x}}_{1}(t)=-\frac{Q_{x\_an}f_{d}\omega_{x\_an}}{k_{x\_an}}\sin\omega_{x\_an}t\\ {\dot{x}}_{2}(t)=\frac{Q_{x\_an}f_{d}\omega_{x\_an}}{k_{x\_an}}\sin\omega_{x\_an}t \end{array}$$

Sense-mode frame:

(3) $$\left\{ \begin{array}{l} m_{s2}{{\ddot{y}}^{\prime }}_{1}+\left(k_{y2}+k_{l2}\right){y^{\prime }}_{1}+k_{y3}\left({y^{\prime }}_{1}-y^{\prime \prime }\right)=f_{l12}\\ m_{s2}{{\ddot{y}}^{\prime }}_{2}+\left(k_{y2}+k_{l2}\right){y^{\prime }}_{1}+k_{y3}\left({y^{\prime }}_{2}+y^{\prime \prime }\right)=f_{l22} \end{array}\right.$$

where $k_{y1}$ is the stiffness of the spring connected to the anchor and the sense-mode frame; $k_{l1}$ represents the stiffness of the spring connected to the sense-mode frame and lever in the sense direction; $k_{y3}$ is the stiffness in the direction of the spring connected to the sense lever and sense-mode frame; and $f_{l11}$ and $f_{l11}$ represent the force exerted on the sense-mode frame by the lever in the left and right tines, respectively.

The leverage mechanism and coordinate relationships are as follows:

(4) $$\left\{ \begin{array}{l} f_{l11}=Bf_{l12}\\ f_{l21}=Bf_{l22}\\ y^{\prime }=By \end{array}\right.$$

where B is the leverage rate (LR), B>1.

Sensing coupling frame:

(5) $$k_{y3}\left({y^{\prime }}_{1}-y^{\prime \prime }\right)=k_{y3}\left({y^{\prime }}_{2}+y^{\prime \prime }\right)$$

According to the above equations, the kinematic analysis of the dual-mass gyroscope in the sense direction can be expressed as:

(6) $$(m_{s1}+m_{c}+B^{2}m_{s2}){\ddot{y}}_{1}+c_{y}{\dot{y}}_{1}+\left(k_{y1}+k_{l1}+B^{2}k_{y2}+B^{2}k_{l2}+\frac{B^{2}k_{y3}}{2}\right)y_{1}+\frac{B^{2}k_{y3}}{2}y_{2}=-2m_{c}\Omega_{z}{\dot{x}}_{1}$$

(7) $$(m_{s1}+m_{c}+B^{2}m_{s2}){\ddot{y}}_{2}+c_{y}{\dot{y}}_{2}+\left(k_{y1}+k_{l1}+B^{2}k_{y2}+B^{2}k_{l2}+\frac{B^{2}k_{y3}}{2}\right)y_{2}+\frac{B^{2}k_{y3}}{2}y_{1}=-2m_{c}\Omega_{z}{\dot{x}}_{2}$$

Subtracting Equation (7) from Equation (6), we obtain

(8) $$(m_{s1}+m_{c}+B^{2}m_{s2})\left({\ddot{y}}_{1}-{\ddot{y}}_{2}\right)+c_{y}\left({\dot{y}}_{1}-{\dot{y}}_{2}\right)+\left(k_{y1}+k_{l1}+B^{2}k_{y2}+B^{2}k_{l2}\right)\left(y_{1}-y_{2}\right)=2f_{c}\sin\omega_{x\_an}t$$

where $f_{c}$ is the Coriolis force at angulate rate Ω*_z_*, which can be given as:

(9) $$f_{c}=\frac{2m_{c}\Omega_{z}Q_{x\_an}f_{d}\omega_{x\_an}}{k_{x\_an}}$$

where $f_{d}$ is the driving force, and $\omega_{x\_an}$, $k_{x}$, and $Q_{x}$ are the resonant frequency, total stiffness, and quality factor, respectively, in the anti-mode.

Adding Equations (6) and (7), we obtain:

(10) $$(m_{s1}+m_{c}+B^{2}m_{s2})\left({\ddot{y}}_{1}+{\ddot{y}}_{2}\right)+c_{y}\left({\dot{y}}_{1}+{\dot{y}}_{2}\right)+\left(k_{y1}+k_{l1}+B^{2}k_{y2}+B^{2}k_{l2}+B^{2}k_{y3}\right)\left(y_{1}+y_{2}\right)=0$$

Since the vibration output of in- and anti-phase modes needs to be explored, a coordinate transformation is made as follows:

(11) $$y_{an}=y_{1}-y_{2},\;y_{in}=y_{1}+y_{2}$$

Substituting Equation (11) into Equations (8) and (9), we obtain:

(12) $$\left\{ \begin{array}{l} {\ddot{y}}_{an}+\frac{\omega_{y\_an}}{Q_{y\_an}}{\dot{y}}_{an}+\omega_{y\_an}^{2}y_{an}=2f_{c}\sin\omega_{x\_an}t\\ {\ddot{y}}_{in}+\frac{\omega_{y\_in}}{Q_{y\_in}}{\dot{y}}_{in}+\omega_{y\_in}^{2}y_{in}=0 \end{array}\right.$$

where $\omega_{y\_an}=\sqrt{\frac{k_{y1}+k_{l1}+B^{2}k_{y2}+B^{2}k_{l2}}{m_{y}}}$, $\omega_{y\_in}=\sqrt{\frac{k_{y1}+k_{l1}+B^{2}k_{y2}+B^{2}k_{l2}+B^{2}k_{y3}}{m_{y}}}$, $Q_{y\_an}=\frac{m_{y}\omega_{y\_an}}{c_{y}}$, $Q_{y\_in}=\frac{m_{y}\omega_{y\_in}}{c_{y}}$, and $m_{y}=m_{s1}+m_{c}+B^{2}m_{s2}$, in which $\omega_{y\_an}$ and $\omega_{y\_in}$ are the defined resonant frequencies and $Q_{y\_an}$ and $Q_{y\_in}$ are the quality factors of the anti- and in-phase motions, respectively, and $m_{y}$ is the total mass in the sense direction.

Equation (12) can be represented as a matrix:

(13) $$M\ddot{y}+C\dot{y}+Ky=F_{c}\sin\omega t$$

where $M=\left[\begin{array}{cc} 1 & \\ & 1 \end{array}\right]$, $C=\left[\begin{array}{cc} \frac{\omega_{y\_an}}{Q_{y\_an}} & \\ & \frac{\omega_{y\_in}}{Q_{y\_in}} \end{array}\right]$, $K=\left[\begin{array}{cc} \omega_{y\_an}^{2} & \\ & \omega_{y\_in}^{2} \end{array}\right]$, $F_{c}=\left[\begin{array}{c} \frac{2f_{c}}{m_{y}}\\ 0 \end{array}\right]$, and $y=\left[\begin{array}{c} y_{an}\\ y_{in} \end{array}\right]$.

The natural frequency can be obtained by using the characteristic equation:

(14) $$\begin{array}{l} \omega_{1}^{2}=\omega_{y\_an}^{2}=\frac{k_{y1}+k_{l1}+B^{2}k_{y2}+B^{2}k_{l2}}{m_{y}}\\ \omega_{2}^{2}=\omega_{y\_in}^{2}=\frac{k_{y1}+k_{l1}+B^{2}k_{y2}+B^{2}k_{l2}+B^{2}k_{y3}}{m_{y}} \end{array}$$

where $\omega_{1}$ and $\omega_{2}$ are the first- and second-order resonant frequency, respectively.

The modal superposition technique is used to acquire the steady-state response by solving Equation (13):

(15) $$y\left(t\right)=\frac{f_{c}\beta_{1}}{m_{y}\omega_{1}^{2}}\left[\begin{array}{c} 1\\ 0 \end{array}\right]\sin\left(\omega_{x\_an}t-\psi_{1}\right)+\frac{f_{c}\beta_{2}}{m_{y}\omega_{2}^{2}}\left[\begin{array}{c} 0\\ 1 \end{array}\right]\sin\left(\omega_{x\_an}t-\psi_{2}\right)$$

where $\beta_{i}=\frac{1}{\sqrt{{\left(1-\lambda_{i}^{2}\right)}^{2}+{\left(\frac{\lambda }{Q_{i}}\right)}^{2}}}$, $\psi_{i}=\arctan\frac{\lambda }{\left(1-\lambda^{2}\right)Q_{i}}$, and $\lambda_{i}=\frac{\omega_{x\_an}}{\omega_{i}}$ are the magnification factors of amplitude, phase angle, and frequency ratio, respectively.

When $\omega =\omega_{x\_an}=\omega_{y\_an}$, we can obtain that:

(16) $$\{\begin{array}{c} {y^{\prime }}_{1}\left(t\right)=By_{an}\left(t\right)=\frac{2m_{c}\Omega_{z}Q_{x\_an}f_{d}\omega BQ_{y\_an}}{k_{y\_\mathrm{an}}k_{x\_an}}\sin\left(\omega t-\frac{\pi }{2}\right)\\ {y^{\prime }}_{2}\left(t\right)=By_{an}\left(t\right)=-\frac{2m_{c}\Omega_{z}Q_{x\_an}f_{d}\omega BQ_{y\_an}}{k_{y\_\mathrm{an}}k_{x\_an}}\sin\left(\omega t-\frac{\pi }{2}\right) \end{array}$$

According to Equation (16), the differential detection output of the tuning fork micromechanical gyroscope is:

(17) $$y_{dA}={y^{\prime }}_{1}\left(t\right)-{y^{\prime }}_{2}\left(t\right)=\frac{2f_{c}BQ_{y\_an}}{k_{y\_\mathrm{an}}}\sin\left(\omega t-\frac{\pi }{2}\right)=\frac{4m_{c}\Omega_{z}Q_{x\_an}f_{d}\omega BQ_{y\_an}}{k_{y\_\mathrm{an}}k_{x\_an}}\sin\left(\omega t-\frac{\pi }{2}\right)$$

The mechanical sensitivity of the type A architecture can be obtained from Equation (17):

(18) $$S_{ma}=\frac{{y^{\prime }}_{1}\left(t\right)-{y^{\prime }}_{2}\left(t\right)}{\Omega_{z}}=\frac{4m_{c}Q_{x\_an}f_{d}\omega BQ_{y\_an}}{k_{y\_\mathrm{an}}k_{x\_an}}\sin\left(\omega t-\frac{\pi }{2}\right)$$

**Figure 3:**
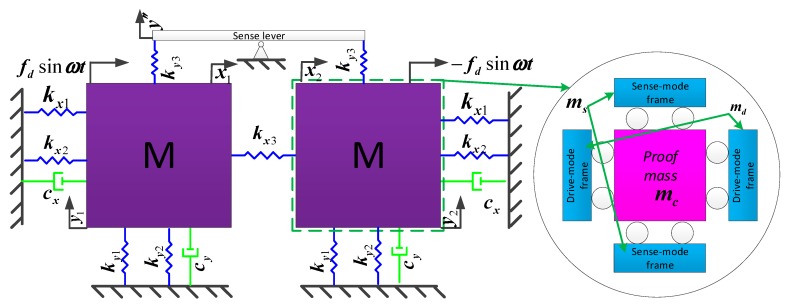
Model of type B.

The 4-dof coupling vibration model of the MEMS gyroscope with equal displacement capacitance detection is shown in Figure 3.

The differential detection output and mechanical sensitivity of the type B architecture can be acquired by the same technique:

(19) $$y_{dB}=y_{1b}\left(t\right)-y_{2B}\left(t\right)=\frac{2f_{c}Q_{yb\_an}}{k_{yb\_\mathrm{an}}}\sin\left(\omega t-\frac{\pi }{2}\right)=\frac{4m_{c}\Omega_{z}Q_{x\_an}f_{d}\omega BQ_{yb\_an}}{k_{yb\_\mathrm{an}}k_{x\_an}}\sin\left(\omega t-\frac{\pi }{2}\right)$$

(20) $$S_{mb}=\frac{4m_{c}Q_{x\_an}f_{d}\omega Q_{yb\_an}}{k_{yb\_\mathrm{an}}k_{x\_an}}\sin\left(\omega t-\frac{\pi }{2}\right)$$

where $Q_{yb\_an}=\frac{m_{yb}\omega_{yb\_an}}{c_{yb}}$, $k_{yb\_an}=k_{y1}+k_{y2}$, $m_{yb}=m_{s2}+m_{c}$, and $\omega_{yb\_an}=\sqrt{\frac{k_{y1}+k_{y2}}{m_{s2}+m_{c}}}$ are the quality factor, total stiffness, total mass, and resonant frequency of the anti-phase mode in the drive direction, respectively.

### 3.2. Optimization Analysis of LR

In order to obtain a more substantial improvement of mechanical sensitivity, it is effective to reduce $k_{y\_an}$ and increase B as much as possible.

Here, the dimensionless parameters α, K, and β are defined in terms of stiffness ratio (SR): α is the stiffness ratio of the power arm (SRPA) and β is the stiffness ratio of the resistance arm (SRRA). The three parameters are given by:

(21) $$\alpha =\frac{k_{l1}}{k_{y1}},\;K=\frac{k_{y2}}{k_{y1}}\;\mathrm{and}\;\beta =\frac{k_{l2}}{k_{y1}}$$

Obviously, tiny LR will lead to an insignificant amplification effect of leverage, which will not be important in truly amplifying the mechanical sensitivity. On the other hand, extremely large LR will increase the detection mode stiffness, thus making it difficult for the tiny Coriolis force to drive the detection mode effectively. Without considering the effect of the quality factor during the design phase, according to Equation (18), we obtain:

(22) $$\frac{\partial S_{ma}}{\partial B}=\frac{\left(1+\alpha \right)-B^{2}\left(K+\beta \right)}{{\left(1+\alpha +B^{2}\left(K+\beta \right)\right)}^{2}}\cdot \frac{4m_{c}Q_{x\_an}f_{d}\omega Q_{y\_an}}{k_{y1}k_{x\_an}}\sin\left(\omega t-\frac{\pi }{2}\right)$$

When $\frac{\partial S_{ma}}{\partial B}=0$, we can obtain that:

(23) $$B=\sqrt{\frac{1+\alpha }{K+\beta }}$$

Equation (23) shows that an optimal solution to this problem exists and different parameters have different effects on the optimal leveraged magnification. Furthermore, the square of B is directly proportional to α and inversely proportional to K and β.

### 3.3. Analysis of IRMS

Type A and type B adopt the same structure and springs, except for the connection between the proof mass and detection frame, and the same fabrication and packaging technique. It is assumed that the damping ratios of the two types of TFG are equal.

The dimensionless parameters η and M are defined by:

(24) $$\eta =\frac{S_{ma}-S_{mb}}{S_{mb}}=\frac{y_{dA}-y_{dB}}{y_{dB}},\;M=\frac{m_{s2}}{m_{c}}$$

where η denotes the improvement rate of mechanical sensitivity (IRMS) and M denotes the mass ratio (MR).

Substituting Equations (18) and (20) into Equation (24), we obtain:

(25) $$\eta =\sqrt{\frac{\left(\left(1+\alpha \right)M+K+\beta \right)\left(1+K\right)}{2\left(1+M\right){\left(K+\beta \right)}^{2}}}-1$$

It can be seen that the IRMS is mainly determined by the stiffness ratios K, α, and β and mass ratio M. By solving the partial derivative of function η with respect to these variables, we obtain that:

(26) $$\frac{\partial \eta }{\partial K}=-\frac{1}{4}\cdot \frac{\left(1+\alpha \right)\left(K-\beta +2\right)M-\sqrt{2}\left(\beta -1\right)\left(K+\beta \right)}{{\left(K+\beta \right)}^{2}\sqrt{\left(\left(1+\alpha \right)M+K+\beta \right)\left(1+M\right)\left(1+K\right)}}$$

(27) $$\frac{\partial \eta }{\partial \alpha }=\frac{\sqrt{2}}{4}\cdot \frac{M\sqrt{1+K}}{\left(K+\beta \right)\sqrt{\left(1+M\right)\left(M\left(1+\alpha \right)+K+\beta \right)}}$$

(28) $$\frac{\partial \eta }{\partial \beta }=-\frac{\sqrt{2}}{4}\cdot \frac{\left(2M\left(1+\alpha \right)+K+\beta \right)\sqrt{1+K}}{{\left(K+\beta \right)}^{2}\sqrt{\left(1+M\right)\left(M\left(1+\alpha \right)+K+\beta \right)}}$$

(29) $$\frac{\partial \eta }{\partial M}=-\frac{\sqrt{2}}{4}\cdot \frac{\left(K+\beta -1+\alpha \right)\sqrt{1+K}}{\left(K+\beta \right)\sqrt{{\left(1+M\right)}^{3}\left(M\left(1+\alpha \right)+K+\beta \right)}}$$

In general, the values of the stiffness ratios K, α, and β and mass ratio M are greater than 0 and less than 1. From Equations (26)–(29), we can find that:

(30) $$\frac{\partial \eta }{\partial K}<0,\;\frac{\partial \eta }{\partial \alpha }>0,\;\frac{\partial \eta }{\partial \beta }<0,\;\frac{\partial \eta }{\partial M}>0$$

From Equation (30), the improvement rate of mechanical sensitivity η monotonically increases with increasing α and M, but decreases with increasing K and β. Within a reasonable range, K and β are as small as possible and α and M are as large as possible. The leverage mechanism can improve the displacement of sense frame effectively and obtain a huge improvement in terms of mechanism sensitivity.

The change of M and K will lead to a corresponding change of the mechanism architecture with no leverage. Furthermore, M and K are system parameters, not just leverage mechanism parameters, which will be analyzed in later studies. Here, four architectures are designed to explore the impact of α and β on IRMS. One is based on type B architecture and the other three are based on type A architecture, defined as types A1, A2 and A3. The $k_{l1}$ of type A2 and the $k_{l2}$ of type A3 are slightly larger than those of type A1. This can be achieved by intentionally increasing the spring width of the leverage mechanism in the sense direction.

## 4. FEM Simulation and Analysis

### 4.1. Analysis of LR and IRMS

**Figure 4:**
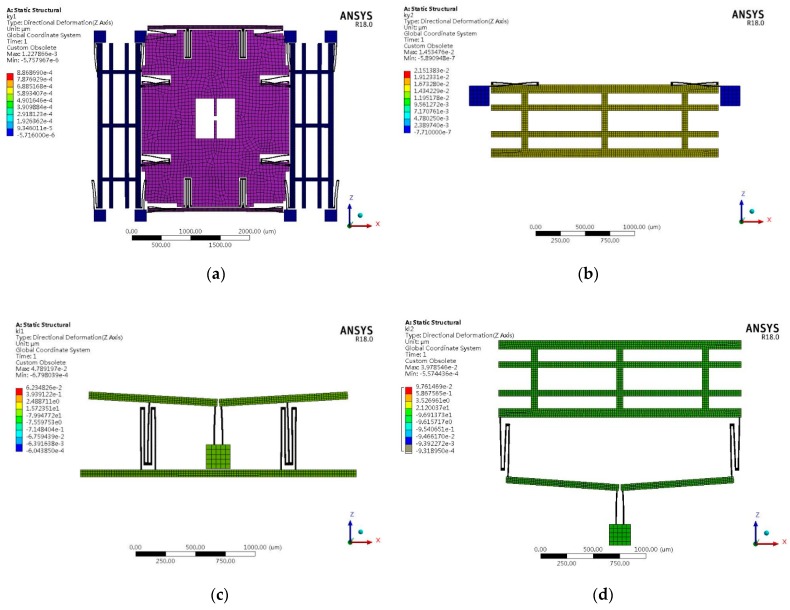
Stiffness of sense mode of micro-electro-mechanical (MEMS) gyroscope: (**a**) deformation of $k_{y1}$; (**b**) deformation of $k_{y2}$; (**c**) deformation of $k_{l1}$; (**d**) deformation of $k_{l2}$.

From Equation (21), the stiffness ratio is dependent on the stiffness of various springs in the sense direction. Therefore, simulations are carried out on the stiffness of these springs by applying a 1 μN force in the linked structure, shown in Figure 4. The length, width and height of the designed spring are 500 μm, 10 μm and 80 μm, respectively. The stiffness of the linear springs connecting the anchor to the proof mass and the sense frame is shown in Figure 4a,b, respectively. The stiffness of the beam associated with the leverage mechanism is shown in Figure 4c,d. Using the formula k=F/x, the stiffness *k* can be obtained:

(31) $$k_{y1}=\frac{1\;\mu N}{0.0012029\;\mu m}=831.32\;N/m,\;k_{y2}=2\cdot \frac{1\;\mu N}{0.0103225\;\mu m}=193.75\;N/m\;k_{l1}=2\cdot \frac{1\;\mu N}{0.025038\;\mu m}=79.88\;N/m,\;k_{l2}=2\cdot \frac{1\;\mu N}{0.039746\;\mu m}=50.32\;N/m\;k_{l1\_2}=2\cdot \frac{1\;\mu N}{0.015593\;\mu m}=128.26\;N/m,\;k_{l2\_3}=2\cdot \frac{1\;\mu N}{0.027567\mu m}=72.55\;N/m$$

Substituting Equation (31) into Equations (21) and (24), we obtain:

(32) $$K=\frac{k_{y2}}{k_{y1}}=\frac{193.75}{831.32}=0.233063,\;\alpha =\frac{k_{l1}}{k_{y1}}=\frac{79.88}{831.32}=0.096088,\;\alpha_{2}=\frac{k_{l1\_2}}{k_{y1}}=\frac{128.26}{831.32}=0.15429\;\beta =\frac{k_{l2}}{k_{y1}}=\frac{50.32}{831.32}=0.06053,\;\beta_{3}=\frac{k_{l2}}{k_{y1}}=\frac{72.55}{831.32}=0.08727$$

Substituting Equation (32) into Equation (23), we obtain:

(33) $$B_{1}=\sqrt{\frac{1+\alpha }{K+\beta }}=\sqrt{\frac{1+0.096088}{0.233063+0.06053}}=1.93,\;B_{2}=\sqrt{\frac{1+\alpha_{2}}{K+\beta }}=\sqrt{\frac{1+0.15429}{0.233063+0.06053}}=1.98\;B_{3}=\sqrt{\frac{1+\alpha }{K+\beta_{3}}}=\sqrt{\frac{1+0.096088}{0.233063+0.08727}}=1.85$$

The mass ratio M is determined by structural design parameters; the mass of the proof mass and sense frame are 1.16e−6 kg and 2.78e−7 kg, so we obtain:

(34) $$M=\frac{m_{s2}}{m_{c}}=\frac{2.774e-7\;\mathrm{kg}}{1.1598e-6\;\mathrm{kg}}=0.23918$$

Therefore, the IRMS of the MEMS tuning fork gyroscope with an anchored leverage mechanism can be calculated from Equations (25), (32) and (34):

(35) $$\eta_{1}=\sqrt{\frac{\left(\left(1+\alpha \right)M+K+\beta \right)\left(1+K\right)}{2\left(1+M\right){\left(K+\beta \right)}^{2}}}-1=79.10\%,\;\eta_{2}=\sqrt{\frac{\left(\left(1+\alpha_{2}\right)M+K+\beta \right)\left(1+K\right)}{2\left(1+M\right){\left(K+\beta \right)}^{2}}}-1=81.33\%\;\eta_{3}=\sqrt{\frac{\left(\left(1+\alpha \right)M+K+\beta_{3}\right)\left(1+K\right)}{2\left(1+M\right){\left(K+\beta_{3}\right)}^{2}}}-1=68.06\%$$

From the above numerical analysis, it is concluded that the IRMS of type A1 is lower than that of type A2 and slightly higher than that of type A3, which is in accord with the theoretical results.

### 4.2. FEM Analysis of Mechanism Sensitivity

#### 4.2.1. Modal Analysis

A structural model simulation is carried out using the ANSYS software. The mesh element is SOLID186, which is a high-order three-dimensional 20-node solid structure unit. All the structures use hexahedral meshes: the linear beam uses a small mesh, and the drive and sense frames use slightly larger meshes. All anchor and proof mass elements use larger hexahedral meshes. Types A1, A2, A3, and B have a total of 543,516, 547,996, 547,039, and 399,356 meshes, respectively. The main parameters of the structures and the material properties of silicon are shown in Table 1 and Table 2, respectively.

**Figure 5:**
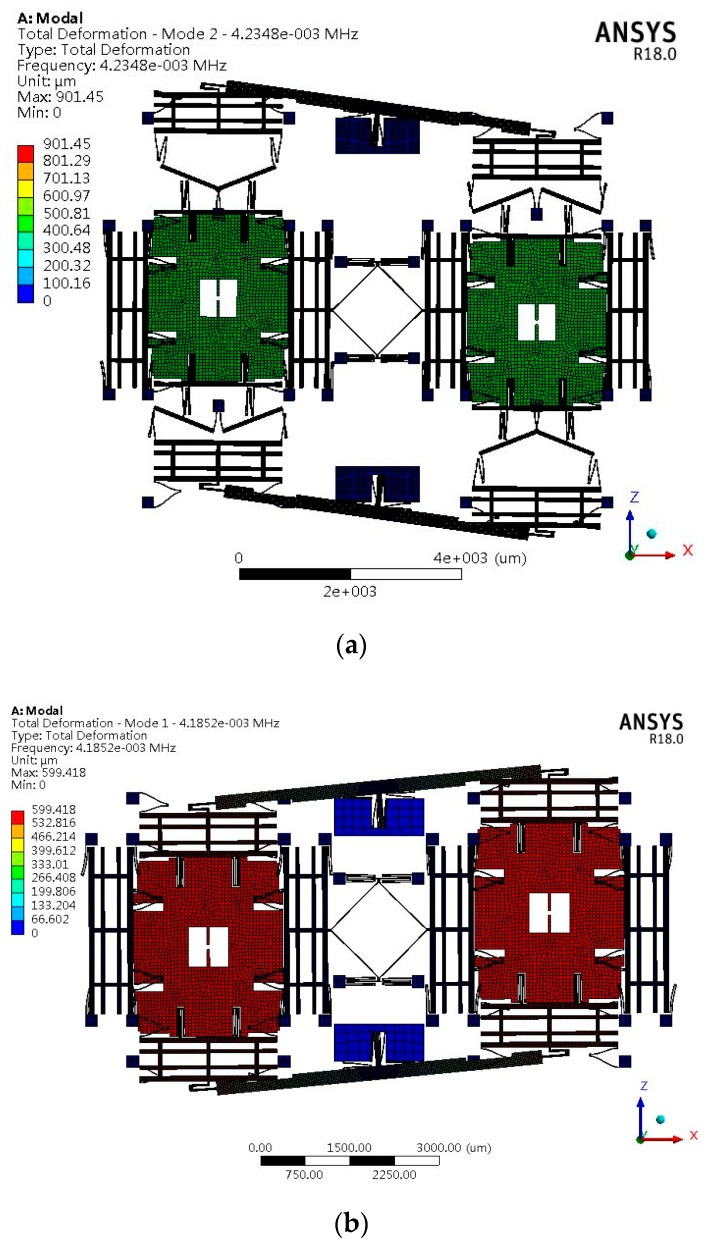
Anti-phase mode frequency in the sense direction of (**a**) type A1 and (**b**) type B.

Figure 5 shows the sense modes of types A1 and B. The anti-sense and anti-drive modes of all types are the first two order modes. The natural frequencies of the first three modes and the corresponding modes of vibration of all types are listed in Table 3.

It can be seen from Table 3 that the anti-sense and anti-drive modes of all types are the first two order modes. The mode order of type A with an anchored leverage mechanism does not change compared to type B. The leverage mechanism can improve the frequency of in-phase sense mode, which can decrease the influence of the common vibration on the mechanical sensitivity.

#### 4.2.2. Sensitivity Analysis

**Figure 6:**
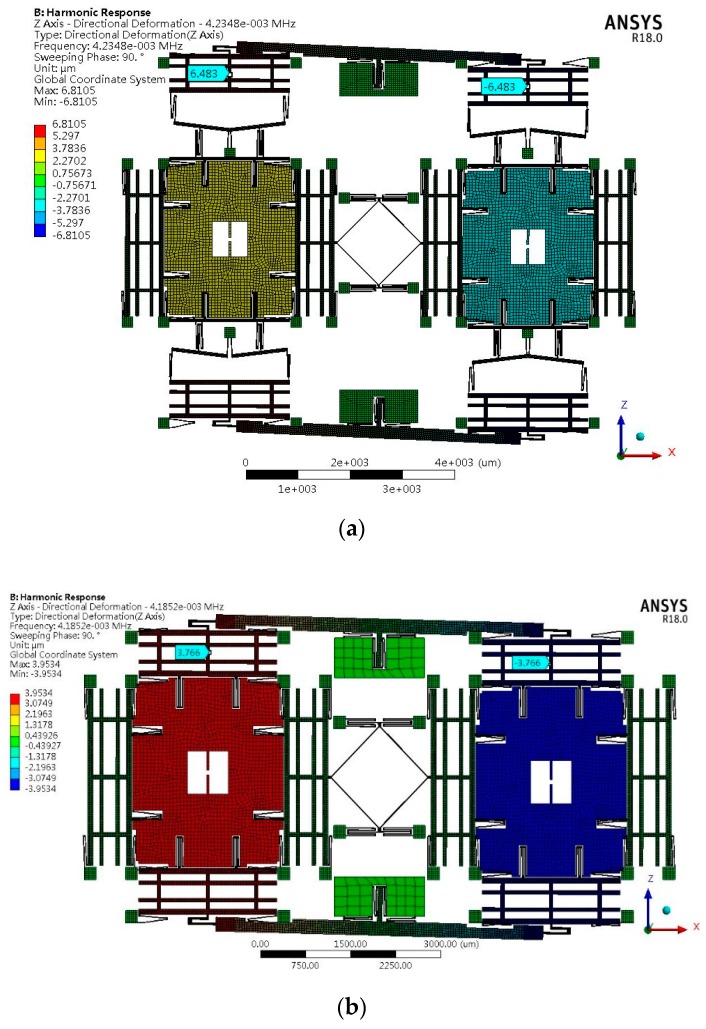
Directional deformation of harmonic response analysis in the sense-mode resonance frequency of (**a**) type A1 and (**b**) type B.

**Figure 7:**
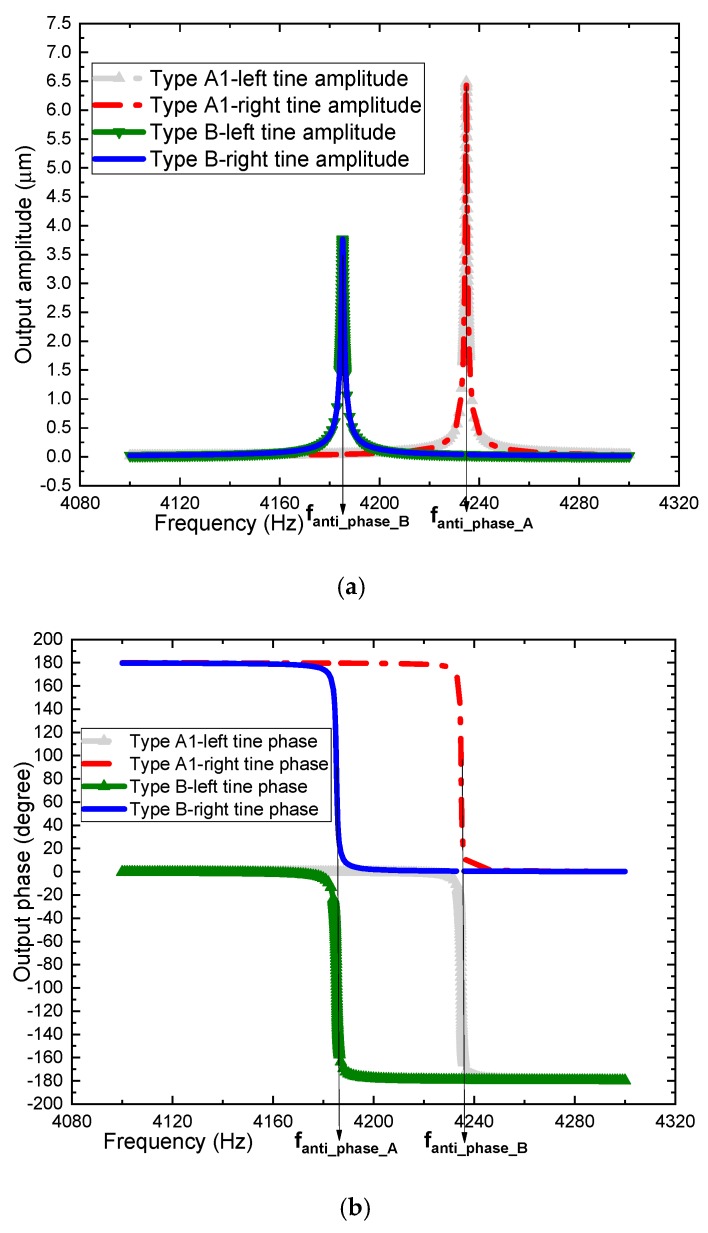
(**a**) Amplitude response and (**b**) phase response of types A1 and B.

To analyze the vibration output response, a mode-based steady-state linear dynamic analysis is performed on the design by ANSYS software to predict the linear response of a structure subjected to continuous harmonic excitation. In this simulation, the parameters of the structure, material properties of silicon, mesh division and element type are the same as those of the modal analysis. According to previous experimental results, the quality factor of MEMS gyroscopes is about 3000–8000 [33]. In this analysis, when the quality factor of type B is 4000, the quality factor of types A1, A2 and A3 can be calculated by the previous assumptions and defined as 6328, 6565 and 6605, respectively. In order to guarantee the accuracy and efficiency of the simulation, the frequency ranges of types A1 and B designs sweep from 4100 Hz to 4300 Hz based on the results of modal analysis in the previous section. The frequency step is 1 Hz. Since the amplitude response around the resonant frequency varies greatly, a harmonic response analysis with higher accuracy needs to be carried out. The frequency range is determined by the previous results. The frequency ranges of types A1 and B designs sweep from 4233.5 Hz to 4236 Hz and from 4184 Hz to 4186.5 Hz, respectively. As a result, the frequency step is 0.025 Hz. In the left and right proof mass of the design, in-phase and anti-phase simple harmonic force with a relative amplitude of 1 μN is applied. The resonance frequency of sense-mode in type A1 and type B is 4234.8 Hz and 4185.2 Hz, respectively, and the vibration amplitude of sense-mode frame is about 6.483 μm and 3.766μm. Figure 6 displays the deformation results at 20 times magnification. The left and right sense mode frame’s amplitude–frequency characteristics of the vibration of the tuning fork structure after loading are shown in Figure 7.

**Figure 8:**
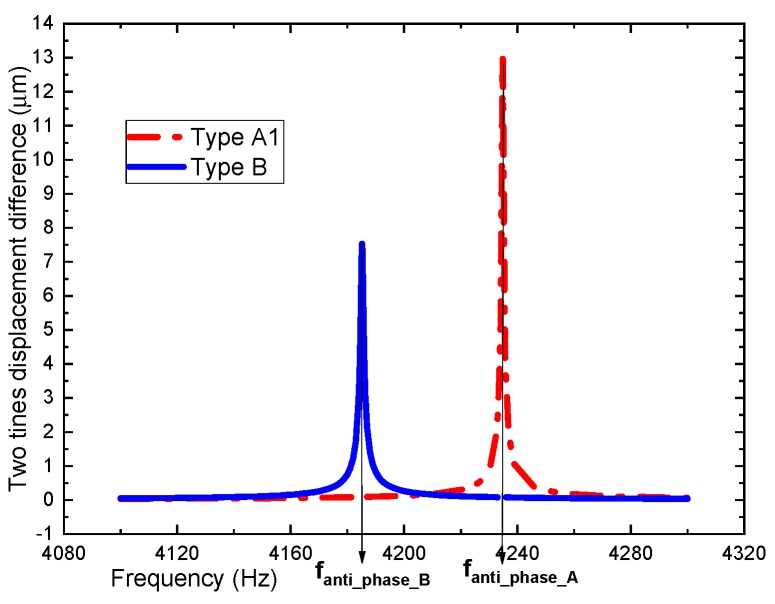
Differential displacement of two tines of types A1 and B.

As depicted in Figure 7, two tines of types A and B have completely synchronized movement to eliminate the vibration output. This verifies the function as a tuning fork. The differential displacement of two tines can be obtained by computing the simulation data with MATLAB software, as shown in Figure 8.

### 4.3. Numerical and Theoretical Comparisons

The differential displacement of two tines can be calculated through the analytical expressions of Equations (17) and (19). The theoretical and simulation values of types A1 and B and the error rate are obtained, as listed in Table 4.

From Table 4, it can be found that the simulation results are consistent with the theoretical values, which verifies the proposed theoretical model. Therefore, the theoretical model can be effective for an anchored leverage mechanism. Meanwhile, the theoretical and simulation values of types A1, A2, and A3 and the error rate are compared in Table 5.

Substituting the simulation data in Table 4 and Table 5 into Equation (25), the IRMS simulation values of types A1, A2, and A3 are obtained. From Equation (35), the theoretical values of the design can be obtained. The comparison of theoretical and simulation values of types A1, A2, and A3 is shown in Table 6.

Table 6 shows that the IRMS of type A1 is lower than that of type A2 and slightly higher than that of type A3 regardless of simulation and theoretical values. It is found that the simulation values of the IRMS are in accord with the theoretical values, although error still exists in these results, which verifies the analysis result of the IRMS in that we proposed that it would increase with increasing α but decrease with increasing β.

### 4.4. Nonlinear Analysis

Scale factor nonlinearity is one of the main errors of MEMS gyroscopes. The structure error and circuit noises are the main causes of nonlinearity of scale factor. [36,37,38]. This paper presents nonlinearity caused by large deformation of elastic beam in ideal TFG designs, other potential errors (non-ideal fabrication, capacitive nonlinearity, etc.) are not covered in this paper.

**Figure 9:**
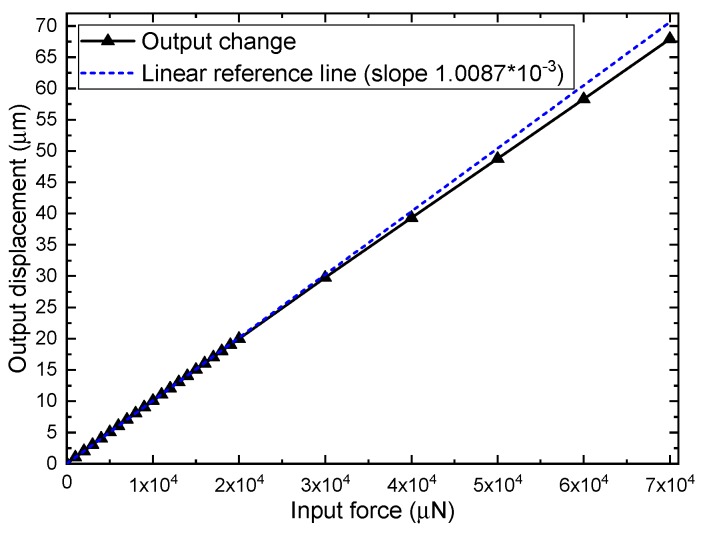
Input force vs. output displacement from 0 μN to $7.0\times 10^{4}\;\mu N$ and linear reference line.

The nonlinear analysis can be used to analyze problems where the stress–strain relationship of the material is nonlinear and check whether the model gives reasonable results. After nonlinear analysis, increasing input force from 0 μN to $7.0\times 10^{4}\;\mu N$ increases displacement of sense-mode frame along *y*-axis from 0 to 67.916 μm as shown in Figure 9, and a linear reference line are used to highlight the changes in stiffness under large deformation. The nonlinearity of the overall structure cannot be ignored when the deflection of elastic beams exceed a certain limit.

**Figure 10:**
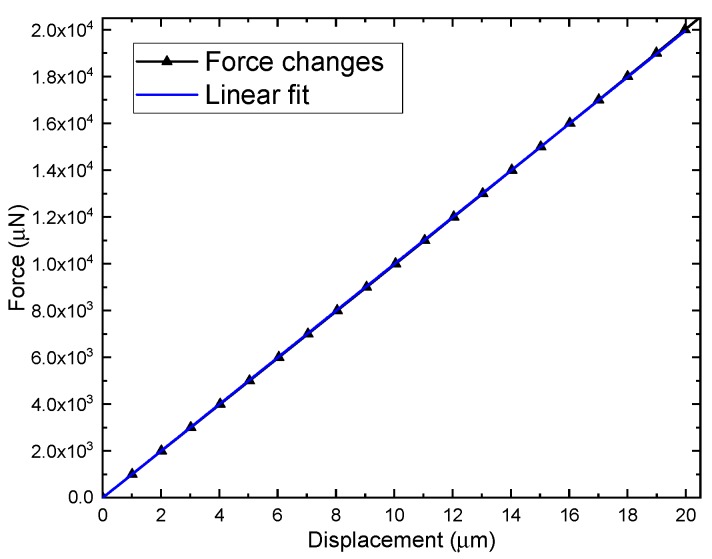
Input force vs. output displacement from 0 μN to $2.0\times 10^{4}\;\mu N$ and linear fit of measured data.

The interval of U-shaped beam in this design is usually relatively small and the width is 20 μm. In addition, solid stoppers should be introduced to improve the shock resistance of the MEMS gyroscope. Therefore, the maximum displacement range of the whole structure is 20 μm. Additionally, to verify the linearity of the TFG in the maximum working range, the deviation from the best-fit line is calculated, as shown in Figure 10. The nonlinearity of the mechanical scale factor is found to be negligible (adjusted R^2^ = 0.999999), which verified that the design works within a linear interval and the validity of the lumped parameter model proposed.

## 5. Discussion

From the theoretical mechanical analysis of a tuning fork gyroscope with anchored leverage mechanism, it is concluded that the IRMS monotonically increases with increasing α but decreases with increasing β. Actually, the stiffness of the springs associated with SR cannot increase or decrease indefinitely. The strength theory of the structure should be considered at the design stage. On the other hand, stoppers, such as elastic or solid stoppers, should be introduced to improve the shock resistance of the MEMS gyroscope.

It is noteworthy that $k_{l1}$ consists of two U-shaped springs in parallel. This structure has the same effect as the decoupling spring so that it can reach the secondary decoupling effect of quadrature couple in the sense direction.

In future studies, the proposed anchored leverage mechanism of the TFG will be fabricated by a traditional silicon-on-glass and deep reactive ion etching process for experimental verification. In addition, it should be noted that sufficient machining accuracy in the lever mechanism is required to ensure consistency of the stiffness of the left and right levers.

## 6. Conclusions

In this paper, a novel MEMS tuning fork gyroscope with an anchored leverage mechanism is presented and a new dynamic model is established to investigate the mechanical sensitivity. Moreover, the leverage rate and improvement rate of mechanical sensitivity are analyzed to represent the optimization effect. The theoretical solutions show that the IRMS monotonically increases with increasing α but decreases with increasing β. Three types of gyro structures with different stiffness ratios are designed. In addition, the stiffness of the springs associated with the stiffness ratio is obtained by an FEM simulation. The IRMS of the design can be calculated as 79.10%, 81.33%, and 68.06%. Finally, modal analysis and harmonic response simulation are carried out. FEM simulation demonstrates that the mechanical sensitivities of the design are in accord with theoretical results, verifying the theoretical model. The linearity of design is analyzed, too. Consequently, the anchored leverage mechanism TFG is confirmed to offer a higher displacement output of sense mode, improving the mechanical sensitivity.

## Figures and Tables

**Table 1:** Main parameters of the structures.

Parameter	Value
Proof mass (${\mu m}^{3}$)	2300×2700×80
Length of drive spring (μm)	440
Width of drive spring (μm)	10
Length of sense spring (μm)	440
Width of sense spring (μm)	10
Length of drive coupling leverage (μm)	1131
Width of drive coupling leverage (μm)	15
Length of sense coupling leverage (μm)	5450
Width of sense coupling leverage (μm)	220
Length of leverage (μm)	1050
Width of leverage (μm)	60
Lever arm length ratio	1.93
Equivalent mass of type A1 (kg)	$2.2469\times 10^{-6}$
Equivalent mass of type A2 (kg)	$2.3019\times 10^{-6}$
Equivalent mass of type A3 (kg)	$2.1604\times 10^{-6}$
Equivalent mass of type B (kg)	$1.4372\times 10^{-6}$

**Table 2:** Material parameters for FEM simulation.

Parameters	Young’s Modulus (Pa)	Poisson’s Ratio	$\mathrm{Density}\;({\mathrm{kg}/m}^{2})$
Values	$1.7\times 10^{11}$	0.28	2330

**Table 3:** Natural frequencies of first three modes and corresponding modes of vibration of all types.

	Order	1	2	3
Type				
Type A1	Frequency (Hz)	4227.3	4234.8	7914.9
	Mode of vibration	Anti-phase of drive	Anti-phase of sense	In-phase of sense
Type A2	Frequency (Hz)	4227.4	4288.9	8068.6
	Mode of vibration	Anti-phase of drive	Anti-phase of sense	In-phase of sense
Type A3	Frequency (Hz)	4227.3	4319.2	7938.4
	Mode of vibration	Anti-phase of drive	Anti-phase of sense	In-phase of sense
Type B	Frequency (Hz)	4185.2	4248	6478.9
	Mode of vibration	Anti-phase of sense	Anti-phase of drive	In-phase of sense

**Table 4:** Comparisons with theoretical and simulation values of types A1 and B.

	Type	Type A1			Type B		
Displacement		Theoretical Value	Simulation Value	Error Rate	Theoretical Value	Simulation Value	Error Rate
Two tines’ displacement difference (μm)		13.418	12.966	3.49%	7.804	7.532	3.62%

**Table 5:** Comparison of theoretical and simulation values of types A1, A2 and A3.

Type	Theoretical Value	Simulation Value	Error Rate
Type A1	13.418	12.966	3.49%
Type A2	13.585	13.098	3.72%
Type A3	12.596	12.141	3.75%

**Table 6:** Comparison of theoretical and simulation values of types A1, A2 and A3.

Type	Theoretical Value	Simulation Value
Type A1	79.10%	72.15%
Type A2	81.33%	73.90%
Type A3	68.06%	61.19%

## References

[1] Shkel A.M. (2006). Type I and type II micromachined vibratory gyroscopes. Proceedings of the IEEE/ION Position, Location, and Navigation Symposium.

[2] Xia D., Yu C., Kong L. (2014). The development of micromachined gyroscope structure and circuitry technology. Sensors.

[3] Perlmutter M., Robin L. High-performance, low cost inertial MEMS: A market in motion!. Proceedings of the IEEE/ION Position Location and Navigation Symposium (PLANS).

[4] Trusov A.A. (2011). Overview of MEMS Gyroscopes: History, Principles of Operations, Types of Measurements.

[5] Guan Y., Gao S., Jin L., Cao L. (2015). Design and vibration sensitivity of a MEMS tuning fork gyroscope with anchored coupling mechanism. Microsyst. Technol..

[6] Guan Y., Gao S., Liu H., Jin L., Niu S. (2016). Design and vibration sensitivity analysis of a MEMS tuning fork gyroscope with an anchored diamond coupling mechanism. Sensors.

[7] Azgin K., Temiz Y., Akin T. An SOI-MEMS tuning fork gyroscope with linearly coupled drive mechanism. Proceedings of the 20th IEEE International Conference on Micro Electro Mechanical Systems (MEMS 2007).

[8] Gomez U.M.K., Kuhlmann B., Classen J., Bauer W., Lang C., Veith M., Frey J., Grabmaier F., Offterdinger K., Raab T. New surface micromachined angular rate sensor for vehicle stabilizing systems in automotive applications. Proceedings of the 13th International Conference on Solid-State Sensors, Actuators and Microsystems.

[9] Weinberg M.S., Kourepenis A. (2006). Error sources in in-plane silicon tuning-fork MEMS gyroscopes. J. Microelectromech. Syst..

[10] Sahin K., Sahin E., Alper S.E., Akin T. (2009). A wide-bandwidth and high-sensitivity robust microgyroscope. J. Micromech. Microeng..

[11] Gando R.K., Kubo H., Masunishi K., Tomizawa Y., Ogawa E., Maeda S., Hatakeyama Y., Itakura T., Ikehashi T. A catch-and-release drive MEMS gyroscope with enhanced sensitivity by mode-matching. Proceedings of the 4th IEEE International Symposium on Inertial Sensors and Systems (INERTIAL).

[12] Xia D., Kong L., Gao H. (2015). A mode matched triaxial vibratory wheel gyroscope with fully decoupled structure. Sensors.

[13] Xu L., Li H., Ni Y., Liu J., Huang L. (2014). Frequency tuning of work modes in *z*-axis dual-mass silicon microgyroscope. J. Sens..

[14] Bu F., Xu D., Zhao H., Fan B., Cheng M. (2018). MEMS gyroscope automatic real-time mode-matching method based on phase-shifted 45 degrees additional force demodulation. Sensors.

[15] He C., Zhao Q., Huang Q., Liu D., Yang Z., Zhang D., Yan G. (2015). A MEMS vibratory gyroscope with real-time mode-matching and robust control for the sense-mode. IEEE Sens. J..

[16] Zaman M.F., Sharma A., Hao Z., Ayazi F. (2008). A mode-matched silicon-yaw tuning-fork gyroscope with subdegree-per-hour allan deviation bias instability. J. Microelectromech. Syst..

[17] Sharma A., Zaman M.F., Ayazi F. (2009). A sub-0.2°/hr bias drift micromechanical silicon gyroscope with automatic CMOS mode-matching. IEEE J. Solid-State Circuits.

[18] Xu L., Li H., Yang C., Huang L. (2016). Comparison of three automatic mode-matching methods for silicon micro-gyroscopes based on phase characteristic. IEEE Sens. J..

[19] Trusov A.A., Schofield A.R., Shkel A.M. (2011). Micromachined rate gyroscope architecture with ultra-high quality factor and improved mode ordering. Sens. Actuators A Phys..

[20] Schofield A.R., Trusov A.A., Shkel A.M. Versatile vacuum packaging for experimental study of resonant MEMS. Proceedings of the 23rd IEEE International Conference on Micro Electro Mechanical Systems (MEMS 2010).

[21] Trusov A.A., Schofield A.R., Shkel A.M. (2008). A substrate energy dissipation mechanism in in-phase and anti-phase micromachined *z*-axis vibratory gyroscopes. J. Micromech. Microeng..

[22] Zotov S.A., Simon B.R., Prikhodko I.P., Trusov A.A., Shkel A.M. (2014). Quality factor maximization through dynamic balancing of tuning fork resonator. Sens. J. IEEE.

[23] Larkin K., Ghommem M., Abdelkefi A. (2018). Significance of size dependent and material structure coupling on the characteristics and performance of nanocrystalline micro/nano gyroscopes. Phys. E Low-Dimens. Syst. Nanostruct..

[24] Zhang Q., Feng L., Cui J., Tang Y., Yao Y. (2018). Design of A New Structure Quartz MEMS Gyroscope with High Sensitivity. IOP Conf. Ser. Mater. Sci. Eng..

[25] Jouaneh M., Yang R. (2003). Modeling of flexure-hinge type lever mechanisms. Precis. Eng..

[26] Xu Q., Li Y. (2011). Analytical modeling, optimization and testing of a compound bridge-type compliant displacement amplifier. Mech. Mach. Theory.

[27] Iqbal S., Malik A.A., Shakoor R.I. (2018). Design and analysis of novel micro displacement amplification mechanism actuated by chevron shaped thermal actuators. Microsyst. Technol..

[28] Iqbal S., Shakoor R.I., Gilani H.N., Abbas H., Malik A.M. (2018). Performance Analysis of Microelectromechanical System Based Displacement Amplification Mechanism. Iran. J. Sci. Technol. Trans. Mech. Eng..

[29] Iqbal S., Malik A., Shakoor R.I. (2018). Kinematic sensitivity analysis of a novel micro-mechanism for displacement amplification. Trans. Can. Soc. Mech. Eng..

[30] Iqbal S., Shakoor R.I., Lai Y., Malik A.M., Bazaz S.A. (2019). Experimental evaluation of force and amplification factor of three different variants of flexure based micro displacement amplification mechanism. Microsyst. Technol..

[31] Zhang J., Su Y., Shi Q., Qiu A.P. (2015). Microelectromechanical resonant accelerometer designed with a high sensitivity. Sensors.

[32] Gao Y., Ding X., Huang L., Li H. (2018). Design and analysis of a novel dual-mass MEMS resonant output gyroscope. AIP Adv..

[33] Gao Y., Huang L., Ding X., Li H. (2018). Design and implementation of a dual-mass MEMS gyroscope with high shock resistance. Sensors.

[34] Cao H., Li H. (2013). Investigation of a vacuum packaged MEMS gyroscope architecture’s temperature robustness. Int. J. Appl. Electromagn. Mech..

[35] Cao H., Li H., Kou Z., Shi Y., Tang J., Ma Z., Shen C., Liu J. (2016). Optimization and Experimentation of Dual-Mass MEMS Gyroscope Quadrature Error Correction Methods. Sensors.

[36] Yoon S.W., Lee S., Najafi K. (2012). Vibration-induced errors in MEMS tuning fork gyroscopes. Sens. Actuators A Phys..

[37] Tang Q., Wang X., Yang Q. (2016). Scale factor model analysis of MEMS gyroscopes. Microsyst. Technol..

[38] Rezaei Kivi A., Azizi S., Khalkhali A. (2015). Sensitivity enhancement of a MEMS sensor in nonlinear regime. Int. J. Mech. Mater. Des..

